# Palpable pediatric thyroid abnormalities – diagnostic pitfalls necessitate a high index of clinical suspicion: a case report

**DOI:** 10.1186/1752-1947-1-29

**Published:** 2007-06-22

**Authors:** Joshua P Klopper, Michael T McDermott

**Affiliations:** 1 Department of Medicine, Division of Endocrinology, Metabolism and Diabetes, University of Colorado at Denver and Health Sciences Center, Aurora, Colorado, USA

## Abstract

A 12-year-old girl presented with a 4 year history of an enlarged, firm thyroid gland. On exam, her thyroid was firm and fixed and an enlarged cervical lymph node was palpable as well. Though a thyroid ultrasound prior to referral was read as thyroiditis, clinical suspicion for thyroid carcinoma mandated continued investigation. The diagnosis of papillary thyroid cancer was established and her workup revealed lymph node metastases as well as a tremendous burden of pulmonary metastases. Pediatric thyroid cancer is extremely rare, but often presents with aggressive disease. Palpable thyroid abnormalities in an individual under 20-years-old should be viewed with suspicion and should be thoroughly investigated to rule out malignancy even in the face of negative diagnostic procedures. Though pediatric papillary thyroid cancer often presents with loco-regional and even distant metastatic disease, mortality rates in follow-up for as long as 20 years are very favorable.

## Background

Thyroid cancer occurring under the age of 20 years is a very rare occurrence with an incidence from 1975–1995 of < 2/100,000 children and adolescents. However, of epithelial derived childhood carcinomas, thyroid cancer is the most common [1]. Similar to adults, papillary thyroid carcinoma is the most common of pediatric thyroid carcinomas representing 85–95% of cases. However, in contrast to adult papillary thyroid carcinoma, pediatric papillary thyroid carcinoma tends to be more aggressive at presentation with a higher incidence of multifocality, neck lymph node disease and extracapsular extension [2,3]. The following case details an aggressive presentation of pediatric papillary thyroid cancer and illustrates the importance of vigilance in the context of an abnormal thyroid physical exam in a child.

## Case Report

A 12 year old female originally from El Salvador was referred to the adult endocrinology clinic for a clinically concerning thyroid gland. Per the patient and her mother's history, her thyroid gland had been found to be enlarged and firm by a physician for at least four years. Several months prior to referral she had a thyroid ultrasound performed at a children's hospital and the report impression was: *"diffusely enlarged echogenic thyroid gland consistent with thyroiditis, no masses"*.

The patient had no complaints. She denied local compressive symptoms or dysphagia. She had no significant past medical history. Her grandmother had surgery for a benign goiter but there was no family history of thyroid cancer and no personal history of head or neck irradiation. She had normal vital signs with a blood pressure of 110/70, pulse of 100, and height of 58 inches with a weight of 92 pounds. Her exam was significant for a diffusely enlarged thyroid gland of approximately 45 grams with an irregular contour and a nodular thickening near the isthmus on the left lobe. The thyroid was extremely firm and fixed. Additionally, she had a 0.5–1 cm firm right sided cervical lymph node near the upper pole of the thyroid. The rest of her physical exam was completely normal. Her TSH was 1.00 mcIU/mL and she had negative anti-thyroid peroxidase and anti-thyroglobulin antibodies. After informed consent was obtained, a fine-needle aspiration biopsy of the left thyroid lobe near the isthmus was performed. The cytology diagnosis was: *"Benign appearing follicular cells*".

**Figure 1:**
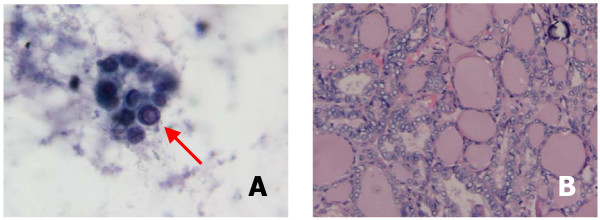
**a**. Cytology consistent with papillary thyroid cancer including nuclear size variation, microfollicle cellular arrangement and an intranuclear pseudo-inclusion (arrow). Papanicolaou stain at 400× magnification. **b**. Final histology showed a papillary carcinoma, follicular variant. There are characteristic nuclear features including ground glass nuclei, inconspicuous nucleoli, thickened nuclear membranes, nuclear pseudoinclusions, and nuclear grooves. This slide also shows a psammoma body. Hematoxylin & Eosin stain at 200× magnification.

The patient was referred for repeat neck ultrasound by an experienced thyroid ultrasound radiologist. He reported: *"A combination of abnormal thyroid gland features (punctate echogenic foci) and the presence of bilateral abnormal lymph nodes with punctate echogenic foci lateral to each internal jugular vein is best explained by multi-focal papillary thyroid cancer and metastases to lymph nodes"*. Based on the ultrasound report with localization of discrete suspicious areas of papillary thyroid cancer within the thyroid gland and abnormally large lymph nodes (some as large as 1.5 cm), the patient was brought back to clinic and repeat thyroid biopsy was performed. A diagnosis of papillary thyroid cancer was made based upon the cytology (fig. 1a).

**Figure 2:**
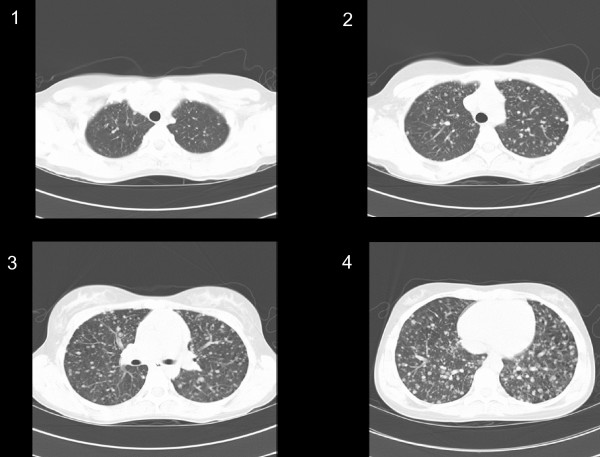
CT scan of the chest that cuts from the apices (1) to the lung bases (4) and shows tremendous tumor burden as well as a basilar prominence of metastases typical of papillary thyroid carcinoma lung metastases.

Prior to near-total thyroidectomy (NTT) and planned radioactive iodine ablation therapy, the patient was sent for a chest x-ray. The chest x-ray showed diffuse miliary nodules throughout both lungs. A non-contrast chest CAT scan was performed and this showed innumerable micronodules throughout both lungs with a preponderance of nodules in the lung bases (fig. 2).

The patient received a near-total thyroidectomy with central neck dissection and modified bilateral neck dissection. During the procedure, the sternohyoid muscles were resected on both sides of the thyroid and a small amount of gross tumor was left on each recurrent laryngeal nerve in lieu of sacrificing those structures. The patient had no voice or breathing complications following the procedure. No muscle groups were removed during the lateral neck dissections. The final histology showed multifocal invasive papillary carcinoma, follicular variant, involving all thyroid lobes with a greatest diameter of 4.7 cm (fig. 1b). There was extracapsular invasion and positive surgical margins with involvement of the thymus noted as well as angiolymphatic invasion. One of three level six central neck lymph nodes were positive for disease as were 9/48 and 9/38 lymph nodes on the left and right neck dissections respectively with no specific delineation of levels two through four lymph node groups. Though only two parathyroid glands were identified on final histology, the patient unfortunately suffered the operative complication of hypoparathyroidism.

**Figure 3:**
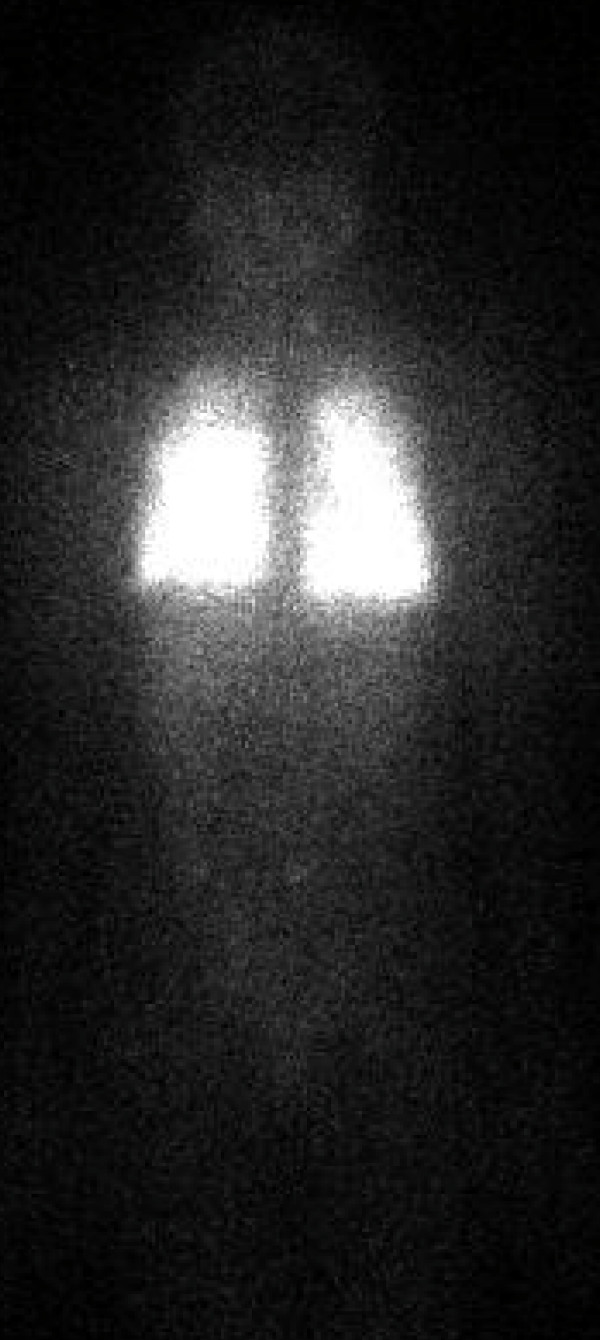
Post-Therapy scan after 150 mCi ^131^I. This anterior view shows tremendous bilateral pulmonary uptake of radioactive iodine, so much so that it visually obliterates the neck uptake detected as well.

In preparing the patient for radioactive iodine ablation, an empiric dose of 200 mCi was considered. With concerns of potential pulmonary fibrosis, dosimetry was performed to assess the maximum dose that would be tolerated without pulmonary damage. Two millicuries of ^131^I were administered orally and 48 hours later the patient returned for a whole body scan (WBS) and uptake analysis in the lungs. The WBS showed multiple foci of uptake bilaterally in the base of the neck and diffuse uptake in the lungs with basilar prominence bilaterally. The stimulated thyroglobulin level was > 13,000 ng/mL indicating a tremendous burden of pulmonary thyroid cancer. The lung uptake was estimated to be 45% and based on geometric mean calculation determined by proprietary software, 150 mCi was considered to be the maximum tolerated dose that would most likely avoid radiation induced pulmonary fibrosis and was given two weeks later. Her post-therapy scan one week after the 150 mCi of ^131^I showed uptake just left of the midline in the base of the neck as well as extensive pulmonary uptake of the radioactive iodine (fig. 3). The patient did not suffer any complications after her radioiodine.

## Discussion

Pediatric thyroid cancer is rare. Risk factors include a family history and previous radiation exposure [4]. The patient presented, however, had no identifiable risk factors. Sporadic papillary thyroid cancer represented only 1.4% of newly diagnosed childhood carcinomas in the USA from 1975–1995 according to the SEER database [5]. Interestingly, the incidence among gender lines changes according to age group with males having a 6:1 increased incidence at ages 5–9, a similar incidence among males and females from ages 10–14 and a ratio more consistent with adult patients with 5:2 female to male ratio after age 14 [5].

Palpable thyroid abnormalities are rare in childhood and should be approached with suspicion and an aggressive evaluation. The incidence of thyroid nodules in children before the onset of puberty is less than two percent in iodine sufficient areas and the aggregate risk of thyroid cancer in a pediatric thyroid nodule can surpass 25% [6]. Our patient had an abnormal thyroid exam for as long as four years prior to being seen in our clinic. The family was unsure of any discrete nodule noted in the past. In this case, the thyroid cancer was diffusely spread through the patient's gland as described and therefore no discrete nodule was present at our initial exam.

The gold standard diagnostic test to define thyroid lesions is fine needle aspiration (FNA) biopsy. Cytological analysis has been evaluated in the pediatric population though the data is somewhat limited by the far less frequent occurrence of pediatric thyroid nodules and FNA biopsies. In a series of 101 specimens from 82 pediatric patients, the diagnostic sensitivity and specificity of detecting carcinoma were 87% and 92% respectively [6,7]. The initial biopsy in this case was read as benign. Based on the concerning clinical features, we simply did not believe that the FNA material was representative of the disease state. It is likely that since there was no discrete nodule the needle simply was not targeted to a malignant portion of the gland. Although an ultrasound guided FNA of an abnormal lymph node would have been an appropriate next step, scheduling difficulties made a second palpation guided FNA after evaluation of the US a better option. Again, the vigilance that is appropriate for pediatric thyroid abnormalities pushed us to continue the pursuit of a clinically suspicious diagnosis. The second FNA confirmed papillary thyroid cancer.

Thyroid ultrasound now plays a prominent and expanding role in the evaluation of thyroid nodules. In a study of patients all under the age of 18 years, thyroid nodule ultrasound features including irregular borders and increased vascular flow assessed by power doppler had positive predictive value for thyroid cancer nodules under 15 mm [8]. The importance of thyroid ultrasound experience cannot be stressed enough in the evaluation of a thyroid gland that is clinically suspicious for a nodule or malignant process. The initial US in this case occurred in a children's hospital where these studies are not routinely performed. Our expert thyroid ultrasound radiologist immediately noted the concerning features for malignancy of echogenic foci (microcalcifications) in the thyroid as well as the same features in enlarged lateral neck lymph nodes [9].

Extensive lymph node disease at the time of diagnosis of pediatric papillary thyroid cancer is not a surprising finding. For adults diagnosed between the ages of 30–60, regional cervical and upper mediastinum lymph node metastases occur in 38–43% of patients [10]. In pediatric patients (age < 21 years old), however, regional nodal metastases occur in 60–80% of cases [3]. We chose to evaluate for distant metastases in this patient's course to help prepare a post-surgical treatment plan. Distant metastases in adult well differentiated papillary thyroid cancer are rare, occurring in only 2–4% of patients at the time of diagnosis [10]. The pediatric population however has distant parenchymal metastases (most often lung) in 10–20% of cases at diagnosis [3].

The assessment of pulmonary metastases prior to radioactive iodine therapy has important implications for choosing a treatment dose and avoiding toxicity, especially pulmonary fibrosis. In children, dosimetry is preferred to assess a safe maximum dose to ideally keep the blood dose of ^131^I < 200–300 cGy and the pulmonary dose to < 80 mCi to avoid pulmonary fibrosis [4]. Our institution does not routinely utilize dosimetry for choosing an adult dose of ^131^I for well differentiated thyroid cancer and we considered giving an empiric dose of 200 mCi to this child. However, dosimetry was appropriately pursued by our nuclear medicine physicians and a dose of 150 mCi was felt to be the safe tolerable dose from a pulmonary safety standpoint.

With such aggressive presentations of pediatric papillary thyroid cancer, one may think that there is excessive mortality. This is not the case. In a retrospective analysis of 14 patients under the age of 17 years diagnosed with sporadic papillary thyroid cancer, all of whom had pulmonary metastases at the time of diagnosis or within 6 months of diagnosis, there was 100% survival over a mean 19 year follow up [2]. Although there is not a high mortality rate, these patients do suffer significant morbidity with a high recurrence rate of their thyroid cancer that requires either multiple surgeries and/or doses of radioactive iodine, as well as long term thyroid hormone suppressive therapy [4].

## Conclusion

Pediatric papillary thyroid cancer can have a very aggressive initial presentation including a high rate of local lymph node metastases and relatively high rate of distant metastases compared to adult patients. Although a lifetime recurrence rate is high, the mortality rates are still low. Palpable thyroid abnormalities in children should be viewed with suspicion and worked up aggressively for possible malignancy even in the face of initially negative diagnostic testing.

## Competing interests

The author(s) declare that they have no competing interests.

## Authors' contributions

JPK primarily evaluated and treated the patient with extensive input and guidance by MTM. JPK primarily drafted the manuscript with input from MTM. All authors read and approved the final manuscript.
